# Racial and Ethnic Disparities in Receipt of Medications for Treatment of COVID-19 — United States, March 2020–August 2021

**DOI:** 10.15585/mmwr.mm7103e1

**Published:** 2022-01-21

**Authors:** Jennifer L. Wiltz, Amy K. Feehan, NoelleAngelique M. Molinari, Chandresh N. Ladva, Benedict I. Truman, Jeffrey Hall, Jason P. Block, Sonja A. Rasmussen, Joshua L. Denson, William E. Trick, Mark G. Weiner, Emily Koumans, Adi Gundlapalli, Thomas W. Carton, Tegan K. Boehmer

**Affiliations:** ^1^CDC COVID-19 Response Team; ^2^Department of Infectious Diseases, Ochsner Clinic Foundation, Jefferson, Louisiana; ^3^Department of Population Medicine, Harvard Pilgrim Health Care Institute, Harvard Medical School, Boston, Massachusetts; ^4^Louisiana Public Health Institute, New Orleans, Louisiana; ^5^College of Medicine and College of Public Health and Health Professions, University of Florida, Gainesville, Florida; ^6^Center for Health Equity and Innovation, Cook County Health, Chicago, Illinois; ^7^Section of Pulmonary Diseases, Critical Care, and Environmental Medicine, Tulane University School of Medicine, New Orleans, Louisiana; ^8^Department of Population Health Sciences, Weill Cornell Medicine, New York, New York.

The COVID-19 pandemic has magnified longstanding health care and social inequities, resulting in disproportionately high COVID-19–associated illness and death among members of racial and ethnic minority groups (*1*). Equitable use of effective medications (*2*) could reduce disparities in these severe outcomes (*3*). Monoclonal antibody (mAb) therapies against SARS-CoV-2, the virus that causes COVID-19, initially received Emergency Use Authorization (EUA) from the Food and Drug Administration (FDA) in November 2020. mAbs are typically administered in an outpatient setting via intravenous infusion or subcutaneous injection and can prevent progression of COVID-19 if given after a positive SARS-CoV-2 test result or for postexposure prophylaxis in patients at high risk for severe illness.^†^ Dexamethasone, a commonly used steroid, and remdesivir, an antiviral drug that received EUA from FDA in May 2020, are used in inpatient settings and help prevent COVID-19 progression^§^ (*2*). No large-scale studies have yet examined the use of mAb by race and ethnicity. Using COVID-19 patient electronic health record data from 41 U.S. health care systems that participated in the PCORnet, the National Patient-Centered Clinical Research Network,^¶^ this study assessed receipt of medications for COVID-19 treatment by race (White, Black, Asian, and Other races [including American Indian or Alaska Native, Native Hawaiian or Other Pacific Islander, and multiple or Other races]) and ethnicity (Hispanic or non-Hispanic). Relative disparities in mAb** treatment among all patients^††^ (805,276) with a positive SARS-CoV-2 test result and in dexamethasone and remdesivir treatment among inpatients^§§^ (120,204) with a positive SARS-CoV-2 test result were calculated. Among all patients with positive SARS-CoV-2 test results, the overall use of mAb was infrequent, with mean monthly use at 4% or less for all racial and ethnic groups. Hispanic patients received mAb 58% less often than did non-Hispanic patients, and Black, Asian, or Other race patients received mAb 22%, 48%, and 47% less often, respectively, than did White patients during November 2020–August 2021. Among inpatients, disparities were different and of lesser magnitude: Hispanic inpatients received dexamethasone 6% less often than did non-Hispanic inpatients, and Black inpatients received remdesivir 9% more often than did White inpatients. Vaccines and preventive measures are the best defense against infection; use of COVID-19 medications postexposure or postinfection can reduce morbidity and mortality and relieve strain on hospitals but are not a substitute for COVID-19 vaccination. Public health policies and programs centered around the specific needs of communities can promote health equity (*4*). Equitable receipt of outpatient treatments, such as mAb and antiviral medications, and implementation of prevention practices are essential to reducing existing racial and ethnic inequities in severe COVID-19–associated illness and death.

The PCORnet-distributed data infrastructure was queried,^¶¶^ and 41 sites*** returned data on monthly receipt of medications for COVID-19 treatment during March 2020–August 2021. The monthly percentage of patients with a positive SARS-CoV-2 test result who received mAb (November 2020–August 2021) and of inpatients with a SARS-CoV-2 positive test result who received dexamethasone or remdesivir (March 2020–August 2021) was calculated separately by race and by ethnicity (as aggregated in PCORnet) for adults aged ≥20 years. Differences in treatment by race and ethnicity were assessed in two ways. First, pairwise Wilcoxon signed rank tests, with p-values indicated as p_w_, were used to assess whether treatment receipt differed systematically over time (systematic temporal differences) by race or ethnicity. Second, relative monthly treatment disparities were calculated as the difference in percentage of patients treated between racial or ethnic minority (Black, Asian, Other for race; Hispanic ethnicity) and majority (White; non-Hispanic) groups divided by the percentage treated in the majority groups for each month.^†††^ The grand means (means of relative monthly treatment disparities) were calculated, and t-tests for statistical difference from zero, with p-values indicated as p_t_, were used to assess presence of overall relative treatment disparities. Results were considered statistically significant for p-values <0.05. GraphPad Prism software (version 9.3.0; GraphPad Software, Inc) was used for analyses and visualization. This activity was reviewed by CDC and conducted consistent with applicable federal law and CDC policy.^§§§^

During March 2020–August 2021, a total of 5,918,199 patients in PCORnet health care systems were tested^¶¶¶^ for SARS-CoV-2, and 805,276 (13.6%) test results were positive (Table 1), representing approximately 3.0% of all positive results reported to CDC (Supplementary Table, https://stacks.cdc.gov/view/cdc/113252). These patients are similar demographically to those included in CDC case data by age, sex, race, and ethnicity. Geographically, patients in the Census Pacific division are underrepresented whereas those in the Mountain division are overrepresented. Among patients with a positive test result, 2.9% were Asian, 15.7% Black, 61.2% White, and 10.9% Other race; 18.6% were Hispanic and 71.7% were non-Hispanic ethnicity (Table 1). Compared with all persons with a positive SARS-CoV-2 test result, a higher proportion of patients with high-risk comorbidities**** were treated with mAb. Critical care^††††^ was required by 3.4% of all persons with positive test results compared with 1.8% of those treated with mAb.

**TABLE 1:** Demographic and medical risk characteristics of patients with positive SARS-CoV-2 test results, by clinical setting and medications received — 41 health care systems in the National Patient-Centered Clinical Research Network, United States, March 2020–August 2021

Characteristic	No. (%)*				
	All patients with positive SARS-CoV-2 test result	Patients receiving monoclonal antibodies	Inpatients with positive SARS-CoV-2 test result	Patients receiving dexamethasone	Patients receiving remdesivir
**No. of unique patients**	805,276	12,539	120,204	40,685	35,315
**Demographics**					
**Age group, yrs**					
20–39	312,680 (38.8)	1,639 (13.1)	20,966 (17.4)	4,966 (12.2)	3,354 (9.5)
40–54	209,202 (26.0)	2,933 (23.4)	23,296 (19.4)	8,285 (20.4)	6,885 (19.5)
55–64	128,550 (16.0)	3,045 (24.3)	24,025 (20.0)	8,874 (21.8)	7,779 (22.0)
65–74	86,848 (10.8)	3,075 (24.5)	24,267 (20.2)	9,124 (22.4)	8,257 (23.4)
75–84	47,047 (5.8)	1,425 (11.4)	18,016 (15.0)	6,420 (15.8)	6,056 (17.1)
≥85	20,949 (2.6)	422 (3.4)	9,634 (8.0)	3,016 (7.4)	2,967 (8.4)
**Sex**					
Female	437,651 (54.3)	6,709 (53.5)	59,583 (49.6)	19,262 (47.3)	16,607 (47.0)
Male	367,359 (45.6)	5,828 (46.5)	60,603 (50.4)	21,416 (52.6)	18,704 (53.0)
Other^†^/Missing^§^	264 (0.0)	3 (0.0)	17 (0.0)	8 (0.0)	3 (0.0)
**Race**					
Asian	22,968 (2.9)	206 (1.6)	4,396 (3.7)	1,219 (3.0)	1,003 (2.8)
Black or African American	126,166 (15.7)	1,904 (15.2)	28,403 (23.6)	8,879 (21.8)	8,172 (23.1)
White	493,181 (61.2)	9,366 (74.7)	59,212 (49.3)	22,910 (56.3)	19,318 (54.7)
Other^¶^	88,026 (10.9)	773 (6.2)	20,729 (17.2)	6,151 (15.1)	5,366 (15.2)
Missing^§^	74,935 (9.3)	280 (2.2)	7,449 (6.2)	1,511 (3.7)	1,443 (4.1)
**Ethnicity**					
Hispanic	149,565 (18.6)	1,006 (8.0)	25,953 (21.6)	7,557 (18.6)	6,895 (19.5)
Non-Hispanic	577,394 (71.7)	11,189 (89.2)	88,007 (73.2)	31,627 (77.7)	27,147 (76.9)
Other**	5,553 (0.7)	20 (0.2)	273 (0.2)	84 (0.2)	104 (0.3)
Missing^§^	72,764 (9.0)	318 (2.5)	5,955 (5.0)	1,410 (3.5)	1,161 (3.3)
**Medical conditions associated with high risk^††^**					
Anemia	72,830 (9.0)	2,187 (17.4)	33,072 (27.5)	9,762 (24.0)	8,553 (24.2)
Arrythmia	73,318 (9.1)	2,527 (20.2)	39,255 (32.7)	12,235 (30.1)	10,828 (30.7)
Asthma	60,080 (7.5)	1,890 (15.1)	16,045 (13.3)	5,301 (13.0)	4,944 (14.0)
COPD	26,636 (3.3)	879 (7.0)	15,330 (12.8)	5,551 (13.6)	5,513 (15.6)
Cancer	37,027 (4.6)	1,641 (13.1)	12,869 (10.7)	4,716 (11.6)	3,605 (10.2)
Chronic kidney disease	50,580 (6.3)	1,795 (14.3)	30,206 (25.1)	9,269 (22.8)	8,418 (23.8)
Chronic pulmonary disorders	100,625 (12.5)	3,219 (25.7)	32,617 (27.1)	11,282 (27.7)	10,582 (30.0)
Coagulopathy	33,374 (4.1)	985 (7.9)	23,070 (19.2)	7,442 (18.3)	6,469 (18.3)
Congestive heart failure	40,179 (5.0)	1,344 (10.7)	24,627 (20.5)	7,868 (19.3)	7,329 (20.8)
Coronary artery disease	54,051 (6.7)	2,074 (16.5)	28,799 (24.0)	9,305 (22.9)	8,607 (24.4)
Diabetes type 2	107,527 (13.4)	3,890 (31.0)	47,963 (39.9)	15,462 (38.0)	14,706 (41.6)
Hypertension	209,848 (26.1)	7,265 (57.9)	78,700 (65.5)	25,653 (63.1)	23,633 (66.9)
Mental health disorders	97,046 (12.1)	2,728 (21.8)	26,443 (22.0)	8,015 (19.7)	7,044 (19.9)
Peripheral vascular disorders	31,930 (4.0)	1,250 (10.0)	16,496 (13.7)	5,373 (13.2)	4,596 (13.0)
Severe obesity (BMI ≥40 kg/m^2^)	60,052 (7.5)	2,430 (19.4)	20,271 (16.9)	7,781 (19.1)	6,891 (19.5)
**Outcome** ^§§^					
Critical care	27,585 (3.4)	225 (1.8)	21,412 (17.8)	10,675 (26.2)	8,244 (23.3)

**Abbreviations**: BMI = body mass index; CDM = common data model; COPD = chronic obstructive pulmonary disease; PCORnet = National Patient-Centered Clinical Research Network.

* Percentages are simple summary numbers (column percentages) out of the total in each category. Strata are not expected to sum to the total because the small cell masking by the data partners before submission of data.

^†^ For sex stratifications, Other includes all remaining PCORnet CDM values that are not male or female.

^§^ For sex, race, and ethnicity stratifications, Missing includes PCORnet CDM values of Refuse to answer, No Information, Unknown, and missing values.

^¶^ For race stratifications, Other includes PCORnet CDM values of Native Hawaiian or Other Pacific Islander, American Indian or Alaska Native, Multiple races, and Other.

** For ethnicity stratifications, Other includes PCORnet CDM values of Other.

^††^ Recorded history of the diagnoses in electronic health record (outpatient or inpatient) within 3 years before a positive test. Patients can have more than one condition.

^§§^ Fourteen days before to 30 days after a positive test result.

Mean monthly mAb use among all patients with positive SARS-CoV-2 test results who were White, Black, Asian, or Other race was 4.0%, 2.8%, 2.2%, and 2.2%, respectively; among patients of Hispanic or non-Hispanic ethnicity, mAb use was 1.8% and 4.0%, respectively. Patients who were Black, Asian, or Other race received mAb 22.4%, 48.3%, and 46.5%, respectively, less often than did White patients (Table 2); systematic temporal differences in mAb receipt were observed by race (all p_w_<0.01) (Figure). SARS-CoV-2 positive patients of Hispanic ethnicity received mAb 57.7% less often (p_t_<0.001) than did non-Hispanic patients; systematic temporal differences in mAb receipt were observed by ethnicity (p_w_ = 0.002).

**TABLE 2:** Average monthly frequency and relative disparity in receipt of medications for treatment of COVID-19, by race and ethnicity — 41 health care systems in the National Patient-Centered Clinical Research Network, United States, March 2020–August 2021

Treatment/Race and ethnicity	Total no. eligible for treatment*	Total no. (%) treated	Mean of monthly percentage treated^†^	p_w_^†^	Mean of monthly relative disparity,^§^ % (95% CI)	p_t_^§^
**Monoclonal antibodies (November 2020–August 2021)**						
**Race**						
White	334,472	9,366 (2.8)	4.0	—	Ref.	—
Black	73,853	1,904 (2.6)	2.8	0.004	−22.4 (−38.7 to −6.1)	0.0125
Asian	14,744	206 (1.4)	2.2	0.002	−48.3 (−63.1 to −33.6)	<0.0001
Other	45,521	773 (1.7)	2.2	0.002	−46.5 (−51.1 to −41.9)	<0.0001
**Ethnicity**						
Non-Hispanic	577,394	11,189 (1.9)	4.0	—	Ref.	—
Hispanic	149,565	1,006 (0.7)	1.8	0.002	−57.7 (−66.6 to −48.9)	<0.0001
**Dexamethasone (March 2020–August 2021)**						
**Race**						
White	59,212	22,910 (38.7)	35.8	—	Ref.	—
Black	28,403	8,879 (31.3)	33.8	0.024	−1.9 (−7.8 to 3.9)	0.498
Asian	4,396	1,219 (27.7)	31.4	0.020	−2.0 (−17.3 to 13.2)	0.782
Other	20,729	6,151 (29.7)	34.2	0.106	−1.3 (−9.1 to 6.6)	0.735
**Ethnicity**						
Non-Hispanic	88,007	31,627 (35.9)	35.4	—	Ref.	—
Hispanic	25,953	7,557 (29.1)	32.5	0.005	−6.2 (−11.7 to −0.6)	0.032
**Remdesivir (March 2020–August 2021)**						
**Race**						
White	59,212	19,318 (32.6)	29.0	—	Ref.	—
Black	28,403	8,172 (28.8)	31.2	0.028	9.3 (0.9 to 17.7)	0.032
Asian	4,396	1,003 (22.8)	26.2	0.200	−15.1 (−30.3 to 0.1)	0.052
Other	20,729	5,366 (25.9)	30.6	0.323	1.7 (−9.4 to 12.8)	0.748
**Ethnicity**						
Non-Hispanic	88,007	27,147 (30.8)	29.3	—	Ref.	—
Hispanic	25,953	6,895 (26.6)	30.4	0.423	8.8 (−0.4 to 18.0)	0.060

**Abbreviation**: Ref. = referent group.

* For monoclonal antibody therapy, all patients with a positive SARS-CoV-2 test result were considered eligible for treatment. For dexamethasone and remdesivir, inpatients with a positive SARS-CoV-2 test result were considered eligible for treatment.

^†^ Mean of monthly treated time series tested for differences using pairwise Wilcoxon signed rank tests with p value given as p_w_. Mean of monthly percent treated = [(n treated / n eligible)_March 2020_ + (n treated / n eligible)_April 2020_ + . . . (n treated / n eligible)_August 2021_] / n total no. months.

^§^ The difference in percentage of patients treated among racial (Black, Asian, or Other races) or ethnic minority (Hispanic) and majority (White or non-Hispanic) groups divided by the percentage treated in the majority groups for each month. Assessed as nonzero using t tests with p-value given as p_t_. Total number of months for dexamethasone and remdesivir = 18 and for monoclonal antibodies = 10. Mean of monthly relative disparity, % = [(Minority − majority / Majority)_March2020_ + (minority − majority / Majority)_April 2020_ . . . + (Minority − majority / Majority)_August 2021_] / Total no. of months.

**FIGURE:**
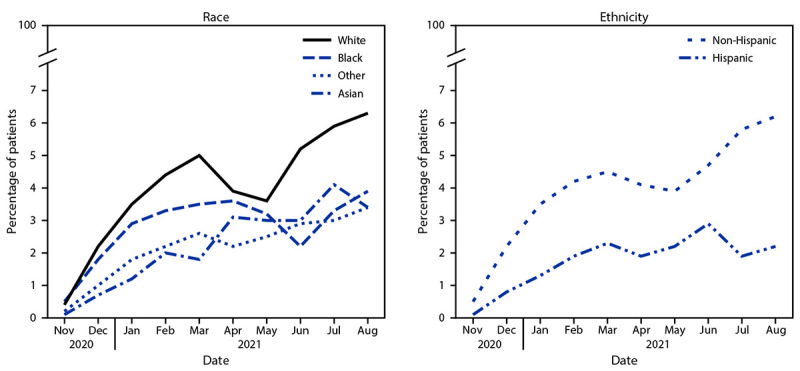
Monthly* percentage of COVID-19 patients (n = 805,276) receiving monoclonal antibody treatment,^†^ by race^§^ and ethnicity^¶^ — 41 health care systems in the National Patient-Centered Clinical Research Network — United States, November 2020–August 2021 * Systematic temporal differences in medication receipt by race and ethnicity were assessed by pairwise Wilcoxon signed rank test. ^†^ mAbs require administration by intravenous infusion or subcutaneous injection. ^§^ White race is the referent group; p-values for Black, Asian, and Other races are 0.004, 0.002, and 0.002, respectively. ^¶^ Non-Hispanic ethnicity is the referent group; p = 0.002 for Hispanic ethnicity.

Mean monthly dexamethasone use among inpatients who were White, Black, Asian, or Other race was 35.8%, 33.8%, 31.4%, and 34.2%, respectively; among patients of Hispanic or non-Hispanic ethnicity, dexamethasone use was 32.5% and 35.4%, respectively. Relative disparities in dexamethasone receipt by race were not statistically significant (Table 2); however, small but systematic temporal differences in dexamethasone receipt were observed among White inpatients and Black, and Asian inpatients (both p_w_<0.05) (Supplementary Figure, https://stacks.cdc.gov/view/cdc/113252). Hispanic inpatients were treated with dexamethasone 6.2% less often than were non-Hispanic inpatients and systematic temporal treatment differences were also observed (p_w_ = 0.005).

Mean monthly remdesivir use among inpatients who were White, Black, Asian, or Other race was 29.0%, 31.2%, 26.2%, and 30.6%, respectively; among patients of Hispanic or non-Hispanic ethnicity, remdesivir use was 30.4% and 29.3%, respectively. Black inpatients received remdesivir 9.3% more often (p_t_ = 0.03) than did White inpatients; systematic temporal differences were also observed (p_w_ = 0.03). Asian, Other race, and Hispanic inpatients did not experience significant relative disparities or systematic temporal differences in remdesivir treatment compared with White and non-Hispanic inpatients.

## Discussion

This large-scale study from 41 U.S. health care systems found disparate mAb treatment of COVID-19 in Hispanic, Black, Asian, and Other race patients relative to non-Hispanic and White patients. Large relative differences were noted for mAb treatment, yet absolute differences were small. Relative differences in treatment with dexamethasone and remdesivir were less apparent in hospital settings, which might be attributed to ease of medication access. mAb treatment must be administered by intravenous infusion or subcutaneous injection by a health care provider, typically in outpatient settings, soon after receipt of a positive test result and within 10 days of symptom onset. The finding of mAb treatment disparities is consistent with previous studies. A single-center study of kidney transplant patients found that Black and Hispanic patients infected with SARS-CoV-2 were less likely to receive mAb and more likely to be hospitalized (*5*). The current study did not identify the underlying causes for the observed disparities. mAb treatment disparities might reflect systemic factors such as limited access to testing and care because of availability constraints, inadequate insurance coverage, and transportation challenges; lack of a primary care provider to recommend treatment; variations in treatment supply and distribution; potential biases in prescribing practices; and limited penetration of messaging in some communities about mAb availability and effectiveness to prevent disease progression. Additional reasons might include hesitancy about receiving treatment; a previous study found patients who were non-Hispanic White and English-speaking accepted mAb treatment more often than did those who were non-White and Hispanic (*6*).

In inpatient settings, Black inpatients received remdesivir more often, and Black, Asian, and Hispanic inpatients received dexamethasone less often than did comparison groups. This could indicate racial and ethnic differences in clinical indications for medication use (e.g., age distribution and prevalence of comorbidities) or could be reflective of varying prescribing practices, protocols, and drug access by institutions that serve populations of different racial and ethnic distributions (*7*).

mAbs are authorized for use in persons at high-risk for severe COVID-19 with positive SARS-CoV-2 test results and as postexposure prophylaxis. In this study, a larger percentage of patients who received mAb had high-risk medical conditions, in accordance with current treatment guidelines. However, this study also found mAb treatments have been used relatively less commonly in racial and ethnic minority groups, amplifying the increased risk for severe COVID-19–associated outcomes, including death among these groups, as a consequence of their higher prevalence of preexisting conditions.^§§§§^

Reducing racial and ethnic disparities in COVID-19 treatment requires patient and clinician awareness of the problem and its solutions; resources; and action from government, private entities, and community- and faith-based organizations to implement effective interventions. Bringing health care to populations facing barriers in access to mAb via a mobile infusion unit or via telehealth providers has been shown to increase mAb use, decrease severe outcomes, and reduce costs (*8*,*9*). These examples of meeting persons in community venues can be helpful in delivering outpatient treatments, addressing pandemic disparities, and managing underlying chronic conditions affected by social determinants of health.^¶¶¶¶^ Moreover, disparities in COVID-19 treatment are the latest example of longstanding unequal treatment of many medical conditions.***** Multicomponent, multisystem programs and policies can support health equity.^†††††^ One such program is the COVID Response and Resilient Communities initiative, which places community health workers in communities to reduce long-standing disparities and deliver interventions to manage COVID-19.^§§§§§^ Future studies of COVID-19 treatment disparities should account for persons with high-risk conditions and include newer medications, such as the oral antiviral agents Paxlovid and molnupiravir, as well as sotrovimab,^¶¶¶¶¶^ which is the only mAb treatment currently available for early treatment of patients infected with the SARS-CoV-2 B.1.1.529 (Omicron) variant.******

The findings in this report are subject to at least five limitations. First, the aggregate data structure did not allow for adjustment of demographic or clinical factors that might be correlated with race and ethnicity. Second, all patients with a positive test result were used as the denominator for calculations of mAb treatment proportions because persons at risk for progression to severe illness could not be identified in aggregate data. Percentage use might be higher and relative disparities might be different if the denominator were specific to mAb prescribing guidelines. Third, missing race and ethnicity was more common among all patients with positive test results than among those treated; more work is needed to fully understand the implications of missing or inaccurate data (*10*). Fourth, mAb use was captured solely from electronic health records; disparities noted here might be restricted to patients who received mAb within a health care system because treatment received in non–health care settings (e.g., government-run infusion sites) is not likely to be recorded. Finally, PCORnet data are derived from a convenience sample of health care facilities, limiting generalizability to the U.S. population.

The COVID-19 pandemic has magnified and amplified inequities that must be addressed to achieve equitable health outcomes. The United States has surpassed 800,000 deaths from COVID-19 and is experiencing another case surge caused by Omicron.^††††††^ Vaccines and preventive measures are the best defense against infection; postinfection, COVID-19 medications reduce morbidity and mortality and relieve strain on hospitals. A lower proportion of persons of racial and ethnic minority groups received mAb outpatient treatment for preventing severe COVID-19. This finding highlights disparities as a priority for intervention and can guide strategies aimed at more equitable COVID-19 outcomes. Policies, resources, and programs addressing the specific needs of served populations, institutions, and places can accelerate progress towards health equity (*4*). Strategizing the equitable receipt of current and emerging outpatient treatments^§§§§§§^ by reducing barriers to accessing treatment might prevent disparities in severe COVID-19 outcomes. Efforts to reduce racial and ethnic disparities with equitable outpatient COVID-19 treatment access, practices, and supportive systems are urgently needed.

**Summary**

**What is already known about this topic?**

Racial and ethnic disparities in SARS-CoV-2 infection risk and death from COVID-19 have been well documented.

**What is added by this report?**

Analysis of data from 41 health care systems participating in the PCORnet, the National Patient-Centered Clinical Research Network, found lower use of monoclonal antibody treatment among Black, Asian, and Other race and Hispanic patients with positive SARS-CoV-2 test results, relative to White and non-Hispanic patients. Racial and ethnic differences were smaller for inpatient administration of remdesivir and dexamethasone.

**What are the implications for public health practice?**

Equitable receipt of COVID-19 treatments by race and ethnicity along with vaccines and other prevention practices are essential to reduce inequities in severe COVID-19–associated illness and death.

## Footnotes

† Fact sheets for healthcare providers for FDA emergency use authorization are available from https://www.fda.gov/media/145611/download for REGEN-COV (casirivimab and imdevimab) and https://www.fda.gov/media/145802/download for bamlanivimab and etesevimab. The SARS-CoV-2 B.1.1.529 (Omicron) variant is not neutralized by bamlanivimab and etesevimab or casirivimab and imdevimab, the mAb-based COVID-19 treatments that were most frequently prescribed before the emergence of Omicron.

§
https://www.covid19treatmentguidelines.nih.gov/management/clinical-management/

¶ PCORnet is a national network-of-networks developed to conduct patient-centered outcomes research. The PCORnet infrastructure supports large-scale studies using its distributed data network. https://doi.org/10.1016/j.jclinepi.2020.09.036

** mAbs included in this study include bamlanivimab, bamlanivimab and etesevimab, casirivimab, and imdevimab, and unspecified monoclonal antibodies. Medications are prescribed or administered in the 14 days before or after the index event.

†† All patients include 78.8% outpatient, 10.9% inpatient, and 10.3% with no associated care setting for mAbs. Care setting was designated with the test.

§§ Care setting was classified as the highest care setting within 16 days of a positive test result but does not necessarily reflect the care setting in which medications were provided. Patients initially tested in the outpatient setting would be assigned to the inpatient setting if they were admitted within 16 days of receipt of a positive test result.

¶¶ A query is a single statistical SAS package that runs at sites to generate the data required. This study used a modular program that generated aggregate data at the site level and combined all results returned to the coordinating center, resulting in a single aggregate report on data across all responding sites.

*** Forty-one sites include Duke University, Medical University of South Carolina, University of North Carolina, Vanderbilt University Medical Center, Wake Forest Baptist Health, Allina Health, Intermountain Healthcare, Medical College of Wisconsin, University of Iowa Healthcare, University of Kansas, University of Nebraska, University of Texas SW Medical Center, University of Utah, University Medical Center New Orleans, Children's Hospital Colorado, Children's Hospital of Philadelphia, Cincinnati Children's Hospital, Nationwide Children's Hospital, Nemours Children's Hospital, Seattle Children's Hospital, St. Louis Children's Hospital, Columbia, Montefiore, Mount Sinai Health System, New York University Langone Medical Center, Weill Cornell Medicine, Lurie Children's Hospital, Northwestern University, Fenway Health, Health Choice Network, OCHIN, Inc, Johns Hopkins University, Ohio State University, Penn State College of Medicine and Penn State Health Milton S. Hershey Medical Center, Temple University, University of Michigan, University of Pittsburgh Medical Center, AdventHealth, Orlando Health System, University of Florida Health, and University of Miami. These sites represent academic and community hospitals; are located across all 50 states, Washington, D.C., Puerto Rico, U.S. Virgin Islands, U.S. Armed forces, and Guam; serve patients who are self-pay, public or privately insured; and total 3.0% of COVID-19 cases (as compared with CDC case surveillance.)

†††
https://pubmed.ncbi.nlm.nih.gov/16032956/

§§§ 45 C.F.R. part 46.102(l)(2), 21 C.F.R. part 56; 42 U.S.C. Sect. 241(d); 5 U.S.C. Sect. 552a; 44 U.S.C. Sect. 3501 et seq.

¶¶¶ Testing was by polymerase chain reaction or antigen test; a positive, detected, or presumptive positive result was considered to be a positive test.

**** High-risk criteria defined by the FDA include age ≥65 years, obesity, pregnancy, chronic kidney disease, diabetes, immunosuppression, cardiovascular disease, and lung disease, along with other underlying conditions that are not explicitly listed in the EUAs for these treatments.

†††† Critical care services are identified by *International Classification of Diseases, Tenth Revision* critical care codes (99291 and 99292) for the evaluation and management of the critically ill or critically injured patient.

§§§§ CDC data on SARS-CoV-2 hospitalization and death by race/ethnicity are available from COVID-NET, a population-based surveillance system collecting data through a network of 250 acute-care hospitals across 14 states (https://www.cdc.gov/coronavirus/2019-ncov/covid-data/investigations-discovery/hospitalization-death-by-race-ethnicity.html). CDC’s National Center for Chronic Disease Prevention and Health Promotion data on 124 chronic disease indicators by race and ethnicity in the U.S. population are available online. https://www.cdc.gov/cdi/index.html

¶¶¶¶
https://www.cdc.gov/chronicdisease/programs-impact/sdoh.htm

*****
https://www.nap.edu/catalog/10260/unequal-treatment-confronting-racial-and-ethnic-disparities-in-health-care

††††† Public health policies and programs centered around the specific needs of communities can promote health equity, including health equity considerations for racial and ethnic minority groups. https://www.cdc.gov/coronavirus/2019-ncov/community/health-equity/race-ethnicity.html

§§§§§ COVID Response and Resilient Communities initiative provides financial support and technical assistance to 69 states, localities, territories, tribes, tribal organizations, urban Indian health organizations, and health service providers to tribes. Intended populations include those at high risk because of their race or ethnicity. https://www.cdc.gov/covid-community-health-workers/pdfs/CCR-fact-sheet-H.pdf

¶¶¶¶¶ Sotrovimab is a mAb authorized for use under an EUA from FDA in May 2021 for the treatment of mild to moderate COVID-19. It was not included in this analysis as it was less commonly used during the study period. The fact sheet for health care providers is available online. https://www.fda.gov/media/149534/download

******
https://www.covid19treatmentguidelines.nih.gov/therapies/statement-on-therapies-for-high-risk-nonhospitalized-patients/

††††††
https://covid.cdc.gov/covid-data-tracker

§§§§§§
https://emergency.cdc.gov/han/2021/han00461.asp
